# Psychometric properties testing of the Myanmar version of the oral health literacy questionnaire among Myanmar migrant mothers of preschool children

**DOI:** 10.3389/fdmed.2026.1871955

**Published:** 2026-08-04

**Authors:** Win Myat Phyo, Naphat Nanthanon, Necharn Leedinchud, Palinee Detsomboonrat, Supassara Chanpakorn

**Affiliations:** 1 International Graduate Program in Dental Public Health, Department of Community Dentistry, Faculty of Dentistry, Chulalongkorn University, Bangkok, Thailand; 2 Doctor of Dental Surgery (DDS) Program, Faculty of Dentistry, Chulalongkorn University, Bangkok, Thailand; 3 Department of Community Dentistry, Faculty of Dentistry, Chulalongkorn University, Bangkok, Thailand

**Keywords:** migrant children, migrant mother, oral health literacy, preschool children, reliability, validity

## Abstract

**Objective:**

To evaluate the validity and reliability of the Myanmar version of the oral health literacy (OHL) questionnaire among Myanmar migrant mothers of preschool children.

**Methods:**

The OHL questionnaire was cross-culturally adapted into the Myanmar language and administered as a self-completed survey among Myanmar migrant mothers of preschool children in Thailand. The questionnaire assessed sociodemographic characteristics, oral health knowledge (OHK), and OHL. Children's dental caries status, assessed using the decayed, missing, and filled teeth (dmft) index and examined independently by two dentists, and mothers' previous dental visits were used to assess discriminant validity. Psychometric properties were evaluated using internal consistency, exploratory factor analysis, and test-retest reliability with the intraclass correlation coefficient (ICC).

**Results:**

A total of 133 mothers participated (mean age 35.2 ± 5.9 years); 46.2% were of Mon ethnicity, 44.6% had a middle school education, and 55.1% were factory workers. Fewer than half understood key dental terms, with the poorest understanding observed for “periodontitis”, “sealants”, and “mottled tooth”, whereas “local anesthesia” was the most well-understood term. Correct knowledge of cariogenic foods (*p* = 0.019), the appropriate age to start toothbrushing (*p* = 0.001), and toothpaste use for preschool children (*p* = 0.002) were significantly associated with OHL scores, while knowledge regarding bottle-feeding cessation and fluoride treatment was not. Exploratory factor analysis identified a two-factor structure consisting of communicative/information OHL and critical evaluation. Inter-examiner reliability for caries assessment indicated good reliability (*κ* = 0.764). The OHL questionnaire demonstrated acceptable internal consistency (Cronbach's *α* = 0.714), but test-retest reliability was poor. The total OHL scores did not differ significantly by children's dental caries experience (*p* = 0.161) and mothers' previous dental visit (*p* = 0.054).

**Conclusion:**

The findings of the present study provide preliminary evidence supporting the internal consistency and two-factor structure of the Myanmar version of the OHL questionnaire; however, further studies are needed to strengthen evidence for its psychometric properties.

## 1 Introduction

Health literacy is a crucial determinant of health and well-being, affecting individuals' ability to access, understand, and utilize health information to make informed health decisions (1). It incorporates both cognitive and social abilities that enable individuals interact with healthcare systems, communicate with healthcare providers, and take part in health-promoting behaviors (2). Oral health literacy (OHL) represents a specific domain within the broader construct of health literacy. It is defined as “the degree to which individuals have the capacity to obtain, process, and understand basic health information and services needed to make appropriate oral health decisions” (3). It plays a critical role in shaping individuals' oral health knowledge (OHK), attitudes, and behaviors, as well as their ability to engage with oral healthcare systems. Individuals with higher OHL are more likely to demonstrate favorable oral health behaviors and outcomes, including the appropriate utilization of dental services, and may benefit from targeted interventions that further enhance OHL. In contrast, individuals with lower OHL tend to exhibit detrimental oral health practices and are at greater risk of oral diseases, such as dental caries (4–6).

Given its importance, the accurate measurement of OHL is essential for identifying populations at risk, informing targeted interventions, and evaluating the effectiveness of oral health promotion programs. Over the past two decades, several instruments have been developed to measure OHL. Early instruments primarily focused on word recognition, reading comprehension, and numeracy, whereas more recent instruments have adopted broader multidimensional approaches that incorporate communication, information processing, and decision-making skills (4, 7). These instruments were originally developed in English and designed to evaluate the most elementary OHL skills including the ability to read, recognize words, and interpret written dental and health-related terms (7). The direct application of such instruments in different populations without appropriate adaptation may compromise their validity and reliability. Language differences can influence the meaning and difficulty of questionnaire items, while cultural factors may affect how individuals interpret health-related concepts and respond to survey questions (8). Therefore, cross-cultural adaptation and validation are essential to ensure that instruments accurately measure OHL in diverse populations. Despite the recognition of this need, important gaps remain in certain populations, particularly those that are socially and linguistically vulnerable.

Migrant populations are frequently underrepresented in health literacy research. Limited OHL may contribute to poorer oral health outcomes within these groups (9). Thailand serves as a major destination for migrant workers from neighboring countries, including Cambodia, Laos, and Myanmar, with approximately 73% of registered migrant workers originating from Myanmar (10). Evidence from vulnerable populations such as refugees and asylum seekers indicates that these groups often experience disproportionately poor oral health outcomes, including a high burden of dental pain and unmet treatment needs, which adversely affect their oral health-related quality of life (11). A systematic review found that individuals with a migration background tended to have lower utilization of dental services, unfavorable oral health beliefs, poorer oral health behaviors, and limited OHK (9). Given its potential impact on OHL as well as on health-related behaviors, attitudes, skills, and beliefs, it is important to consider individual's cultural and ethnic backgrounds in dental education and the design of oral health promotion programs (9).

Mothers or primary caregivers are crucial to managing health-related decisions in the family, including accessing healthcare services and evaluating health information. Their ability to grasp and use oral health information is necessary for communicating with healthcare providers and accessing health services (12, 13). Therefore, the availability of a valid and reliable instrument to evaluate OHL of parents or primary caregivers is essential for identifying individuals who may require additional support or educational interventions. However, evidence regarding OHL among Myanmar parents remains limited. A recent qualitative study among Myanmar migrant parents in Thailand reported limited knowledge of primary teeth, reliance on social media as a major source of oral health information, variability in the ability to evaluate the reliability of oral health information, and several barriers to oral health service utilization (14). These findings suggest heterogeneity in OHL among Myanmar migrant parents and support the need for further research using validated instruments to assess OHL in this population.

Although many instruments exist to measure OHL, no single instrument is available as a gold standard for OHL measurement (7). According to Nutbeam, health literacy can be categorized into three domains: functional literacy, encompassing basic reading skills necessary for comprehending health-related information; communicative or interactive literacy, involving advanced skills that enable individuals to actively engage in healthcare interactions; and critical literacy, which refers to the ability to evaluate information and make informed decisions (15). A new OHL questionnaire developed among Thai mothers of preschool children was designed based on this framework, incorporating these three domains as proposed by Nutbeam, and thus provide a comprehensive approach to measuring OHL (13). Since the questionnaire was originally developed and validated among Thai mothers of preschool children, its applicability to Myanmar migrant populations cannot be assumed because linguistic and cultural differences may influence how questionnaire items are interpreted and answered. As such, cross-cultural adaptation and psychometric evaluation are necessary before the instrument can be used in Myanmar migrant populations. Therefore, the aim of this study was to cross-culturally adapt and evaluate the psychometric properties, including validity and reliability, of the Myanmar version of the OHL questionnaire originally developed among Thai mothers of preschool children.

## 2 Materials and methods

### 2.1 Study design

A cross-sectional study was conducted to perform cross-cultural adaptation and psychometric evaluation of the Myanmar version of OHL questionnaire originally developed among Thai mothers of preschool children. This study was performed in compliance with the Declaration of Helsinki and received approval from the Human Research Ethics Committee (HREC-DCU 2025–075) of the Faculty of Dentistry, Chulalongkorn University, Bangkok, Thailand.

### 2.2 Sample size calculation

The sample size was calculated based on the Cronbach's alpha coefficient-estimation using a web-based sample size calculator (https://wnarifin.github.io/ssc/ssalpha.html) (16). The calculation assumed an expected Cronbach's alpha value of 0.80, a precision of 5% and a 95% confidence level, resulting in a minimum required sample size of 131 participants. A 10% non-response rate was considered; therefore, 146 participants were invited to participate in this study.

### 2.3 Study population

This study was conducted among Myanmar migrant mothers of 5-year-old preschool children in Samut Sakhon Province. Data were collected in July 2025 from the participants recruited across five public schools enrolling Myanmar migrant children in the province. A convenience sampling approach was adopted to facilitate data collection within the study's logistical and time constraints. Participants were eligible if they were Myanmar migrant mothers of preschool children who had lived in Samut Sakhon Province for at least six months and self-reported that they were able to read and understand the Myanmar language sufficiently to complete a written questionnaire. The residency criterion was applied to ensure that participants were familiar with local health services and health information resources available in the province. In this study, “Myanmar migrant” refers to participants whose country of origin was Myanmar, and the questionnaire was administered in the official language of Myanmar. Eligibility was confirmed during recruitment prior to questionnaire administration. Mothers were excluded if they had cognitive or communication impairments that limited completion of the questionnaire, were not the primary caregivers of their preschool children, or were planning to relocate outside Thailand during the study period. Written informed consent was obtained from all participants prior to participation.

### 2.4 Clinical examination

Two trained dentists performed clinical examinations in school settings, with the children seated in a supine position on portable dental chairs under an external light source. A sterilized plain mouth mirror and a Community Periodontal Index (CPI) probe were used to assess dental caries status using the decayed, missing, and filled teeth (dmft) index, according to the criteria described in the World Health Organization oral health survey methods (17). All clinical records were documented electronically via the Tooth Memo application by experienced research assistants (18). Inter-examiner reliability was assessed by re-evaluating 10% of the participants (*n* = 13).

### 2.5 Cross-cultural adaptation

After obtaining permission from the original developer, the Myanmar version of the OHL questionnaire was developed. The original Thai version of OHL questionnaire was translated into the Myanmar language by two independent bilingual translators who were fluent in Thai and Myanmar languages and were working as professional medical interpreters, in accordance with established cross-cultural adaptation guidelines (19). The translated version was then reviewed by an expert panel to evaluate conceptual and item equivalence, resulting in a preliminary draft. The expert panel consisted of a pediatric dentist, a dental public health specialist, and a researcher with expertise in oral health research and questionnaire development. The preliminary draft was subsequently back-translated into Thai language by two other independent bilingual translators, fluent in both Thai and Myanmar languages and working as professional medical interpreters, who were blinded to the original version. The expert panel compared the back-translated version with the original questionnaire to assess semantic consistency and developed a revised second draft based on their evaluation. The preliminary Myanmar version of the OHL questionnaire was then pilot tested among 10 Myanmar migrant parents to evaluate its clarity and comprehensibility. Feedback from the pilot testing was reviewed by expert panel and used to make minor wording refinements to improve clarity and readability. No major conceptual changes to the questionnaire were required. Following these revisions, the final Myanmar version of the OHL questionnaire was established and approved for the psychometric evaluation among Myanmar migrant mothers.

### 2.6 OHL questionnaire

The Myanmar version of the OHL questionnaire consists of four parts: (i) sociodemographic information, including seven questions on age, ethnicity, education, occupation, income, sources of oral health information, and frequency of dental visits; (ii) OHK test, comprising five questions on cariogenic foods, toothbrushing practice, bottle feeding, the use of fluoride toothpaste, and fluoride treatment for preschool children; (iii) basic/functional OHL test consisting of 20 items assessing word reading and comprehension across topics such as dental anatomy, pathology, treatment, and oral health instructions, as well as five additional items measuring understanding of consent forms and post-operative instructions; and (iv) communicative/information OHL and critical evaluation, comprising ten items that access the ability to evaluate the reliability of information and apply it effectively in practice, using a five-point Likert scale with scores ranging from 1 (strongly disagree) to 5 (strongly agree).

### 2.7 Statistical analysis, validity and reliability

All data were analyzed using IBM SPSS software version 29.0.2.0 (SPSS Inc., Chicago, USA) with the level of statistical significance set at 0.05. The normality of the data distribution was evaluated using the Kolmogorov–Smirnov test. Agreement between the two dentists in diagnosing dental caries was assessed using Cohen's kappa coefficient. Descriptive statistics were used to summarize participants' characteristics and study variables.

In psychometric evaluation, validity reflects the degree to which an instrument measures the intended construct and supports the interpretation of its scores. Common forms of validity include content validity, criterion validity, and construct validity. Reliability reflects the consistency, stability, and reproducibility of measurements across repeated administrations and different measurement conditions. Reliability is commonly assessed through internal consistency, test-retest reliability, inter-rater reliability, and measurement error (20). In this study, construct validity was evaluated by examining discriminant validity, which was assessed by comparing OHL questionnaire scores according to (i) dental caries experience, measured using dmft index, and (ii) mothers' previous dental visit (never, only when experiencing symptoms, or routine visits). Children were categorized into two groups: caries-free (dmft = 0) and those with caries experience (dmft > 0). Exploratory factor analysis was performed to identify the underlying structure of the questionnaire. Factors were extracted using principal component analysis, and an Oblimin rotation with Kaiser Normalization was applied to achieve a clearer factor solution. Items with factor loadings below 0.40 were excluded, while those with loadings ≥ 0.40 were retained for interpretation.

The reliability of the Myanmar version of the OHL questionnaire was evaluated in terms of internal consistency and test-retest reliability. Internal consistency was assessed using the Cronbach's alpha coefficient. A Cronbach's alpha value of ≥0.70 was considered acceptable (21). Test-retest reliability was assessed to determine the stability of the questionnaire over time. Seven percent of the participants (*n* = 9) completed the questionnaire twice, with a one-month interval. Agreement between the two measurements was evaluated using the Intraclass Correlation Coefficient (ICC) and ICC values were interpreted as follows: <0.50 (poor), 0.50–0.75 (moderate), 0.75–0.90 (good), and >0.90 (excellent reliability) (22).

## 3 Results

Of the 146 participants invited, 133 Myanmar migrant mothers, with a mean age of 35.2 ± 5.9 years, agreed to participate in the study, resulting in a participation rate of 91.1%. Most participants were of Mon (46.2%) and Bamar (41.0%) ethnicities. Majority had completed middle school education (44.6%) and were employed in the factory (55.1%). Over half of the families reported a monthly income of ≤10,000 THB (56.9%). The internet was the most common source of oral health information (49.3%), followed by television or radio (15.3%). More than half of the mothers had never visited a dentist (52.3%), while 35.4% reported seeking dental care only when symptomatic. The mean dmft score of children was 8.76 ± 5.46, and inter-examiner reliability for caries assessment was good (Cohen's kappa = 0.764) (Table 1).

**Table 1:** Sociodemographic information of mother participants and caries status of their children.

Characteristics of mother participants		*n* (%)
Age (years) (*n* = 133)	mean ± SD	35.2 ± 5.94
Ethnicity (*n* = 132)	Bamar	54 (41%)
	Karen	14 (10.6%)
	Mon	61 (46.2%)
	Other	3 (2.3%)
Education (*n* = 130)	No formal education	18 (13.9%)
	Primary school education	26 (20%)
	Middle school education	58 (44.6%)
	High school education	24 (18.5%)
	Bachelor's degree	3 (2.3%)
	Other	1 (0.8%)
Occupation (*n* = 127)	Agriculture	2 (1.6%)
	Food service	3 (2.4%)
	Factory	70 (55.1%)
	Construction	9 (7.1%)
	Marine/Fishery industry	11 (8.7%)
	Cleaning service	12 (9.4%)
	Sales	9 (7.1%)
	Office work	2 (1.6%)
	Own business	2 (1.6%)
	Other	7 (5.5%)
Income (*n* = 130)	<10,000 THB	74 (56.9%)
	10,001–15,000 THB	38 (29.2%)
	15,001–20,000 THB	10 (7.7%)
	20,001–25,000 THB	2 (1.5%)
	25,001–30,000 THB	3 (2.3%)
	30,001–35,000 THB	2 (1.5%)
	35,001–40,000 THB	1 (0.8%)
Source of oral health information (*can choose more than one*) (all answers=150)	Television/ Radio	23 (15.33%)
	Friends/ Relatives	24 (16%)
	Newspaper/ Magazine	1 (0.67%)
	Dental health/ health personnel	24 (16%)
	Internet	74 (49.33%)
	Brochure/ Poster	2 (1.33%)
	Other	2 (1.33%)
Previous dental visit (*n* = 130)	Never	68 (52.3%)
	Only when having symptoms	47 (36.2%)
	Routinely (once/year)	11 (8.5%)
	Routinely (twice/year)	4 (3.1%)

Children's caries status		mean ± SD
dmft (*n* = 133)	dt	8.35 ± 5.234
	mt	0.4 ± 0.998
	ft	0.01 ± 0.87
	dmft	8.76 ± 5.458

The sample size varies across variables because of missing responses. Percentages were calculated based on the number of valid responses for each variable.

The results of the basic/functional OHL test are presented in Table 2. Overall participants demonstrated relatively low levels of correct responses across many items. In the domain of anatomical terminology, the proportion of correct responses ranged from 28.6% for “pulp” to 36.8% for “root canal”, indicating limited understanding of basic dental anatomy. Similarly, in the pathology domain, correct responses were generally low, particularly for “periodontitis” (5.3%) and “mottled tooth” (10.5%), while slightly higher recognition was observed for “gingival recession” (34.6%). In the treatment domain, knowledge varied considerably across items. The highest proportion of correct responses was observed for “local anesthesia” (69.2%), whereas knowledge of “sealants” was notably low (9.0%). Other treatment-related items, such as “root canal treatment” (12.8%) and “dental bridges” (18.0%), also showed limited understanding. Regarding oral hygiene instruction, correct responses were moderate for “correct toothbrushing technique” (39.8%), “oral examination” (38.3%), and “toothpaste label” (41.4%), but lower for “correct flossing technique” (19.5%) and “toothbrushing and flossing for the prevention of gingivitis” (11.3%). In the information understanding domain, participants demonstrated better comprehension of patient rights and post-extraction instructions, with correct responses of 45.9% for “consent form” and 48.9% for “post-operative instructions”. However, recognition of abnormal symptoms after tooth extraction remained low (12.0%). Overall, these findings indicate that while some basic concepts are relatively well understood, substantial gaps remain in participants' basic/functional OHL, particularly in relation to dental pathology and specific treatment procedures.

**Table 2:** Basic/functional oral health literacy test (*n* = 133).

Basic/functional oral health literacy			Correct responses, *n* (%)
Assessment	Category	Item	
Wording comprehension	Anatomy	Pulp	38 (28.6%)
		Enamel	44 (33.1%)
		Dentine	42 (31.6%)
		Root canal	49 (36.8%)
		Alveolar bone	46 (34.6%)
	Pathology	Tooth shift	41 (30.8%)
		Gingival recession	46 (34.6%)
		Mottled tooth	14 (10.5%)
		Dental plaque	32 (24.1%)
		Periodontitis	7 (5.3%)
	Treatment	Dental bridges	24 (18.0%)
		Local anesthesia	92 (69.2%)
		Sealants	12 (9.0%)
		Root canal treatment	17 (12.8%)
		Scaling	46 (34.6%)
	Oral hygiene instruction	Correct toothbrushing technique	53 (39.8%)
		Correct flossing technique	26 (19.5%)
		Toothbrushing and flossing for the prevention of gingivitis	15 (11.3%)
		Oral examination	51 (38.3%)
		Toothpaste label	55 (41.4%)
Information understanding	Patient's right	Consent form	61 (45.9%)
		Claim	32 (24.1%)
	Post-operative care following tooth extraction	Instruction	65 (48.9%)
		Normal symptom	39 (29.3%)
		Abnormal symptom	16 (12.0%)

Table 3 presents the mean OHL scores according to participants' OHK. Participants who correctly identified the appropriate time to start toothbrushing for their children had significantly higher OHL scores (38.8 ± 5.22) compared with those who answered incorrectly or did not know (35.32 ± 5.36; *p* = 0.001). Similarly, participants who had correct knowledge regarding toothpaste use for preschool children demonstrated higher OHL scores (37.96 ± 5.14) than those with incorrect knowledge (34.96 ± 5.47; *p* = 0.002). In contrast, for cariogenic food, participants with correct responses had significantly lower OHL scores (34.65 ± 6.29) compared with those who answered incorrectly or did not know. (37.00 ± 4.89; *p* = 0.019). However, no significant differences in OHL scores were observed for knowledge regarding the appropriate time to stop bottle feeding (*p* = 0.287) or the benefits of fluoride treatment (*p* = 0.081) although mean OHL scores were slightly higher among participants with correct knowledge. Overall, these findings suggest that higher OHL is associated with better knowledge in certain domains; however, this relationship is not consistent across all knowledge areas.

**Table 3:** Mean and standard deviation of mothers’ oral health literacy (OHL) score by oral health knowledge (OHK) responses.

Oral health knowledge (OHK)		Number of responses, *n* (%)	OHL score, mean (SD)	*p*-value
Question	Response			
Cariogenic food (*n* = 133)	Correct	87 (65.4%)	34.65 (6.29)	0.019*
	Not correct/ Do not know	46 (34.6%)	37 (4.89)	
When to start brushing for children (*n* = 131)	Correct	35 (26.3%)	38.8 (5.22)	0.001*
	Not correct/ Do not know	96 (72.2%)	35.32 (5.36)	
When to stop bottle feeding (*n* = 133)	Correct	30 (22.6%)	37.13 (6.2)	0.287
	Not correct/ Do not know	103 (77.4%)	35.91 (5.3)	
Toothpaste for preschool children (*n* = 133)	Correct	55 (41.4%)	37.96 (5.14)	0.002*
	Not correct/ Do not know	78 (56.8%)	34.96 (5.47)	
Benefit of fluoride treatment (*n* = 133)	Correct	40 (30.1%)	37.49 (5.539)	0.081
	Not correct/ Do not know	93 (69.9%)	35.65 (5.469)	

The sample size varies across variables because of missing responses. Percentages were calculated based on the number of valid responses for each variable.

* *p* < 0.05.

Exploratory factor analysis identified a two-factor structure for the communicative/information OHL and critical evaluation questionnaire (Table 4). Factor 1 comprised items Q1, Q2, Q4, and Q5, representing the domain of communicative/information OHL, while Factor 2 included items Q6, Q7, Q8, Q9, and Q10, corresponding to critical evaluation. All items demonstrated acceptable factor loadings, ranging from 0.617 to 0.818 for Factor 1 and from 0.745 to 0.899 for Factor 2. The highest loading within Factor 1 was observed for Q1 (0.818), while Q7 showed the highest loading for Factor 2 (0.899). Communalities ranged from 0.489 to 0.782, indicating that a substantial proportion of variance in each item was explained by the extracted factors. Item Q3 (loading = 0.28) was removed, while items with loadings ≥ 0.40 were retained. The results support a clear and interpretable two-factor structure consistent with the conceptual domains of communicative/information OHL and critical evaluation.

**Table 4:** Factor loadings for the communicative/information and critical evaluation factors in oral health literacy questionnaire.

Statement	Factor		Communalities
	1	2	
Q1. If you notice that your child has dental caries, you begin to seek relevant information.	0.818		0.569
Q2. When you receive information about dental caries, you are able to select the information you need.	0.704		0.542
Q4. When you receive information about dental caries, you are able to explain it to others in a way they can understand.	0.617		0.489
Q5. When you receive information about how to take care of your child's teeth, you apply the information in your daily life.	0.751		0.613
Q6. When you receive oral health information, you are able to decide which information is appropriate for you		0.821	0.743
Q7. When you receive oral health information, you are able to decide which information is reliable.		0.899	0.782
Q8. When you receive oral health information, you are able to verify that the information is accurate and trustworthy.		0.745	0.707
Q9. When you receive oral health information, you are able to filter out the information which is not reliable.		0.855	0.723
Q10. If you notice that your child has dental caries, you are able to seek information that supports decision-making for practical actions.		0.859	0.673

Factor 1: Q1, Q2, Q4, Q5 addressing “Communicative/information OHL”.

Factor 2: Q6, Q7, Q8, Q9, Q10 addressing “Critical evaluation”.

***Q3 was removed due to a low factor loading (0.28), which was below the predefined threshold of 0.40.

Extraction and rotation method: Principal Component Analysis, Oblimin with Kaiser Normalization.

Table 5 presents the mean OHL scores across different domains according to children's caries status. Overall, there were no statistically significant differences in total OHL scores between caries-free children (dmft = 0) and those with caries (dmft > 0) (38.29 ± 4.57 vs. 36.19 ± 5.49, *p* = 0.161). Similarly, no significant differences were observed in OHK domain (*p* = 0.290), basic/functional OHL (*p* = 0.488), or communicative/information OHL (*p* = 0.294), although mean scores tended to be slightly higher among children with caries free in some domains. However, a statistically significant difference was observed in the critical evaluation domain, where mothers of caries-free children demonstrated higher scores (14.57 ± 1.13) compared with those whose children had caries (13.19 ± 2.45; *p* = 0.009). These findings suggest that while overall OHL may not differ significantly by caries status, the mothers' ability to critically evaluate oral health information may be associated with lower caries experience in children.

**Table 5:** Mean score and standard deviation of mothers’ oral health literacy (OHL) in each domain according to children's caries status.

OHL questionnaire domains (score range)	OHL score, mean ± SD		*p*-value
	Caries-free (dmft = 0, *n* = 7)	With caries (dmft > 0, *n* = 124)	
Oral health knowledge (0–5)	1.43 ± 0.79	1.90 ± 1.16	0.290
Basic/functional OHL (0–25)	7.14 ± 4.18	6.94 ± 4.05	0.448
Communicative/information OHL (4–20; items Q1, Q2, Q4, Q5)	16.57 ± 0.98	16.06 ± 2.45	0.294
Critical evaluation (5–25; items Q6–Q10)	14.57 ± 1.13	13.19 ± 2.45	0.009*
Total OHL score (9–75)	38.29 ± 4.57	36.19 ± 5.49	0.161

The discriminant validity analysis was based on participants with complete OHL and dmft data (*n* = 131).

* *p* < 0.05.

Table 6 presents the mean OHL scores across different domains according to mothers' previous dental visit patterns. Significance differences were observed only for the basic/functional OHL domain (*p* < 0.001). Mean basic/functional OHL scores increased across the three groups, from mothers who had never visited a dentist (5.62 ± 3.64) to those who visited only when experiencing symptoms (7.94 ± 4.23) and those who attended routine dental visits (9.33 ± 3.60). No significant differences were found among the groups for OHK (*p* = 0.254), communicative/information OHL (*p* = 0.984), critical evaluation (*p* = 0.222), or the total OHL score (*p* = 0.054). Although the mean total OHL score was highest among mothers who attended routine dental visits (41.00 ± 4.77), the difference did not reach statistical significance.

**Table 6:** Mean score and standard deviation of mothers’ oral health literacy (OHL) in each domain according to previous dental visits .

OHL questionnaire domains (score range)	OHL score, mean ± SD			*p*-value
	Never (*n* = 68)	Only when having symptoms (*n* = 47)	Routinely (*n* = 15)	
Oral health knowledge (0–5)	1.74 ± 1.07	1.93 ± 1.24	2.27 ± 1.16	0.254
Basic/functional OHL (0–25)	5.62 ± 3.64	7.94 ± 4.23	9.33 ± 3.60	<0.001*
Communicative/information OHL (4–20; items Q1, Q2, Q4, Q5)	16.04 ± 2.42	16.19 ± 2.45	16.13 ± 2.23	0.948
Critical evaluation (5–25; items Q6–Q10)	13.59 ± 2.26	12.79 ± 2.73	13.27 ± 2.02	0.222
Total OHL score (9–75)	37.09 ± 5.67	38.87 ± 6.89	41.00 ± 4.77	0.054

The discriminant validity analysis was based on participants with complete OHL and previous dental visit data (*n* = 130).

* *p* < 0.05.

The internal consistency of the questionnaire was assessed using Cronbach's alpha and the overall Cronbach's alpha for the OHL instrument was 0.714, indicating acceptable internal consistency. The Cronbach's alpha coefficients for the individual domains were 0.238 for OHK, 0.729 for basic/functional OHL, 0.820 for communicative/information OHL, and 0.925 for critical evaluation, indicating varying levels of internal consistency across domains. The low internal consistency observed for the OHK domain may be attributable to the small number of items and the heterogeneous nature of the knowledge constructs assessed, suggesting that the items may not represent a single homogeneous construct. In contrast, the high Cronbach's alpha observed for the critical evaluation domain indicates strong inter-item consistency. However, the possibility that some items assess closely related aspects of the construct cannot be excluded. Test-retest reliability was assessed in a subset participant (*n* = 9) who completed the questionnaire twice with a one-month interval. The ICC for the overall OHL score was 0.016 (95% confidence interval: −0.495–0.352), indicating very low stability over time. Given the small retest sample and the wide confidence interval, this estimate should be interpreted with caution.

## 4 Discussion

This study reports on the cross-cultural adaptation process and the validation of the Myanmar version of the OHL questionnaire among Myanmar migrant mothers of preschool children in Thailand. Overall, the findings indicate that the adapted OHL questionnaire in Myanmar language demonstrated acceptable internal consistency and a clear and interpretable two-factor structure, although limitations were observed in its test-retest reliability.

The Myanmar version of the OHL questionnaire evaluated in the present study differs from other OHL instruments in its conceptual foundation and scope. While instruments such as the Rapid Estimate of Adult Literacy in Dentistry (REALD-30), REALD-99, and Oral Health Literacy Assessment in English (OHLA-E) primarily assess on word recognition and reading comprehension (23), other instruments, including the Oral Health Literacy Instrument (OHLI), the Health Literacy in Dentistry scale (HeLD), and the Oral Health Literacy Adults Questionnaire (OHL-AQ), have expanded OHL assessment beyond simple word recognition. The OHLI incorporates reading comprehension and numeracy skills (24), while the HeLD adopts a broader perspective by assessing multiple dimensions of OHL including communication, access to care and information, understanding, information use, social support, receptivity, and financial barriers (25). The OHL-AQ further extends assessment by evaluating functional OHL, listening, and decision-making skills (26). Unlike these instruments, the questionnaire used in the present study was developed in accordance with Nutbeam's multidimensional health literacy framework and assesses functional, communicative/information, and critical evaluation OHL domains. This broader conceptual approach may provide a more comprehensive assessment of OHL among Myanmar migrant mothers.

The exploratory factor analysis in this study identified a two-factor structure comprising communicative/information OHL and critical evaluation. In contrast, the original Thai questionnaire demonstrated a three-factor structure, including critical OHL, communicative OHL, searching and using information (13). The reduction in the numbers of factors observed in the present study may suggest overlapping constructs within this population, particularly between communicative and information-related domains. This finding may reflect differences in linguistic adaptation, cultural context, and participants' familiarity with oral health information. Cross-cultural studies commonly report such variations in factor structure, as health literacy is a multidimensional and context-dependent construct influenced by social, cultural and environmental factors (15). These findings support the need for contextual validation when applying OHL questionnaire across different populations.

The internal consistency of the Myanmar version of the OHL questionnaire was acceptable (Cronbach's alpha = 0.714), which is comparable to the original Thai version (Cronbach's alpha = 0.76) (13). This indicates that the questionnaire items were sufficiently correlated and may reflect a coherent construct. The interpretation of the test-retest reliability was limited by the small retest sample. In the present study population of migrant workers, repeated follow-up was challenging due to work schedules and limited availability, which contributed to the small retest sample. Consequently, the reliability estimates may become unstable, and confidence in the observed results is limited. In addition, the relatively long interval between assessments may have influenced participants' responses, particularly if exposure to new information or experiences altered their understanding. Given that OHL may change over time, especially among migrant populations adapting to new environments, variability in responses is plausible. Further studies should consider retention strategies suitable for migrant workers and evaluate test-rest reliability using a larger retest sample and an appropriate follow-up interval.

The results of the basic/functional OHL assessment indicated generally low levels of correct responses, particularly in domains related to dental pathology and treatment procedures. This finding is consistent with previous evidence suggesting that migrant populations may have lower levels of OHL (9). The limited basic/functional OHL observed among Myanmar migrant mothers in this study may be related to factors such as limited familiarity with dental terminology, restricted exposure to oral health information, and lower educational attainment. Notably, nearly half of the participants in this study had only completed middle school education, which may be associated with difficulties in understanding and applying oral health information.

The association between OHK and OHL observed in this study was similar to the original Thai version (13). Participants with correct knowledge in certain domains demonstrated higher OHL scores, supporting the relationship between OHK and OHL. However, this relationship was not consistent across all knowledge items, and an inverse association was observed for cariogenic food knowledge. This inconsistency may reflect differences in item complexity, guessing effects, or uneven exposure to oral health information. It also suggests that OHL may encompass not only knowledge but also skills related to interpretation, evaluation and application of information.

No statistically significant association was observed between total OHL scores and children's caries status. However, a significant difference was found in the critical evaluation domain, with higher scores among mothers of children without caries. Previous studies have reported association between parental OHL and children's oral health outcomes, with lower OHL being associated with poorer oral health behaviors and a higher prevalence of dental caries (6, 27). In addition, mothers who reported routine dental visits had a significantly higher basic/functional OHL scores than those who had never visited a dentist. This finding suggests that mothers with more regular dental attendance tend to have higher basic/functional OHL, possibly reflecting greater exposure to oral health information through dental services. However, the lack of significant differences in other domains indicates that routine dental visits alone may not be sufficient to enhance more advanced aspects of OHL, such as critical evaluation and communication skills. These findings provide preliminary evidence supporting the discriminant validity of the Myanmar version of the OHL questionnaire, as specific OHL domains were able to distinguish between groups expected to differ in oral health outcomes and oral health behaviors. However, no significant differences were observed for the total OHL score in either comparison. Therefore, the questionnaire appears to demonstrate greater discriminative ability at the domain level than at the overall score level. These findings should be interpreted cautiously because of the small number of participants in the caries-free group and the routine dental visit group, and this imbalance was not predefined by the study design but reflected the high caries burden in this vulnerable migrant population.

Some limitations should be considered when interpreting the findings of this study. First, the test-retest reliability assessment was based on a small number of participants, limiting confidence in the estimated temporal stability of the questionnaire. Second, the discriminant validity analyses were constrained by the very small number of caries-free children and mothers reporting routine dental visits, as well as the imbalance between comparison groups, which may have limited the robustness of the between-group comparisons. Third, detailed language-background information, including primary language, language at home, and preferred language for health information, was not collected. Therefore, potential associations between language background and questionnaire performance could not be examined. Fourth, although participants were required to be able to read and understand the Myanmar language, the findings may not be generalizable to all ethnics or linguistics groups originating from Myanmar. Finally, the factor structure identified in the present study was derived from exploratory factor analysis. Although the questionnaire demonstrated acceptable psychometric properties, future studies using larger samples should employ confirmatory factor analysis to further examine and validate the proposed factor structure. Despite these limitations, this study addresses an important gap by providing a culturally adapted Myanmar-language OHL questionnaire for use among Myanmar-speaking populations. The lack of validated OHL instruments for migrant populations may limit efforts to understand and address their oral health needs. The questionnaire demonstrated acceptable psychometric properties and may serve as a useful tool for future research assessing OHL among Myanmar-speaking populations. It may also support the development and evaluation of targeted oral health promotion programs and interventions for this underserved group.

## 5 Conclusion

In conclusion, the Myanmar version of the OHL questionnaire demonstrated acceptable internal consistency and a clear and interpretable two-factor structure among Myanmar migrant mothers. Although test-retest reliability was poor and evidence for discriminant validity was limited, the questionnaire provides a potentially useful instrument for assessing OHL among Myanmar migrants who can read and understand Myanmar language. Further studies with larger samples are recommended to strengthen the evidence for its psychometric properties and evaluate its association with oral health outcomes.

## Data Availability

The original contributions presented in the study are included in the article/Supplementary Material, further inquiries can be directed to the corresponding author/s.
